# Hospital Administration: Finance

**Published:** 1892-08-20

**Authors:** Richard Kershaw

**Affiliations:** Central Throat and Ear Hospital


### HOSPITAL ADMINISTRATION.—FINANCE.

Mr. Richard Kershaw, Central Throat and Ear Hospital, writes:—The editorial footnote attached to the publication of my letter in your issue of the 6th inst. compels me to ask the favour of your inserting a rejoinder. Referring to your points *seriatum*:—

1. It is gratifying to find that you are in accord with me on the only point I raised, that it is impossible to compare a small special hospital with a large general hospital, for the purposes of gauging the average cost of each occupied bed, unless the out-patients are taken into account.

2. You appear to sneer at this hospital because with our 16 beds at command we can only boast of 12 in continuous occupation; but I may inform you that in the past year these 16 beds have afforded accommodation to 288 in-patients, and that in the past ten years 2,429 in-patients have been treated, showing an average of 242 per annum. With such figures as these, where, I should like to know, is the mistake in designating such an establishment by the name of hospital?

3. Speaking for the 6,450 out-patients who attended here, it is but fair to assume that they could not have been equally well treated at the general hospitals, or such a large proportion would not have been sent specially to us by members of the medical profession.

4. Your remark that "the estimated expense of the out-patients (3s. 9d. a head) is excessive when compared with the slight outlay of the department," is surely made in great ignorance of the work we are called upon to do, and your ideas have been based on the cost of out-patients at general hospitals and dispensaries, where the patients one after the other require nothing beyond a bottle of medicine or lotion, and that largely from what is commonly known as the "stock jar." At this hospital we have special lime-light illumination and an extensive array of special surgical instruments to keep up, and in one prescription it is often necessary to give two or three kinds of local treatment besides constitutional. The cost of drugs and surgical instruments alone amounted last year to £433 1s. 6d. Nevertheless, by publishing this letter you will place on record the fact that the cost of each out-patient at this hospital, although it is 3s. 9d., is actually less than that of 25 dispensaries which receive a grant from the Hospital Sunday Fund, while our per centage of cost of management to that of maintenance is much less than that of 10 general hospitals, 36 special hospitals, and 19 dispensaries. Indeed, none of the general hospitals can claim a monopoly of economy of management over that of the special hospitals.

---

It is only natural that colour-blindness should not have attained industrial importance till the introduction of railways and the vast increase of shipping. These two factors could not fail to bring science to bear on the subject, though it is only of recent years that science can be said to have taken the matter in hand seriously and systematically. The Royal Society's investigations on the subject are now placed before the public in the form of a Blue Book, which contains much of interest, not only to marine and railway people, but to the world in general. It will be, perhaps, well for smokers to bear in mind that defective colour vision may be of two kinds, one which is a matter of formation and is unalterable, and a second which is the result of disease. This latter is most frequently caused by the excessive use of tobacco. It has been asserted that experiments have proved that no other colours can be used instead of red and green as signal lights, chiefly on account of the diminished luminosity and visibility of the colours which have been proposed as substitutes. This statement is controverted by a correspondent in the *Times*, who is colour-blind, and to whom, and to almost all, as he believes, who are colour-blind, the distinction between red or green and blue is unmistakeable. But the value of blue as a contrasting colour to green or red depends upon the strength of its luminosity and visibility, which, according to the experiments made, is insufficient.

